# Summer Recreations

**Published:** 1869-06

**Authors:** 


### HALL'S JOURNAL OF HEALTH.

VOL. XVI. JUNE, 1869. No. 6.

### SUMMER RECREATIONS

should be adapted to the habits and needs of the individual, in order to obtain the highest amount of health upon which to begin another year's work in the autumn. The brain-racked

BANKER, STOCK-DEALER AND FINANCIER, require a very different kind of recreation from the automatic book-keeper, the hard-working mechanic and the tread mill clerk.

THE WOMAN OF FASHION

could not possibly exist where the care-worn mother of a growing family would find her heaven; nor would the student or professional man obtain the recreation which he needs, in places where retired merchants and pursy bondholders would find their happiness.

Those who seek pleasure in a round of dances and fashionable frolic, where the german and the horse-race, the card-table and the drive, are the order of the day, will gather to the sea side and the spa; they will seek Newport, the maelstrom of money; or Long Branch with its broiling sun and its retinue of demimondes and stuck-up-ities; or Saratoga with its clouds of dust, its gaming houses, its race fields and its disgusting waters which nobody ever quaffed without a grimace and hopes, Oh how deferred!

Mothers who are burdened with the perplexities of housekeeping and the responsibilities of growing families, will find rest for the body, quiet for the mind, and health for both, by seeking the retired farm-house, many miles away from railroad stations and steamboat landings and stage coach stopping places; where with perfect exemption from household cares and fashionable calls and ceremonial visits, she can eat and drink and dress and sleep and read or exercise, at times and seasons perfectly convenient to the bodily instinct and mental inclinations and preferences; where the only preparation needed for a wholesome walk, is the throwing on of the old-fashioned "sun bonnet;" and where you are never doomed to the up-hill work, so up-hilly! of having to talk for politeness' sake, when you can't for your life think of a single word that it would be appropriate to utter. Few know what recreation is; it is literally a "making over," procuring a new supply of vigor to the brain and strength to the body. Nothing can give mental elasticity but sleep; nothing so well gives strength to the hard-working body as muscular rest.

THE MECHANIC

and those who are on their feet a great deal, need not go on fishing excursions, or hunting or boatings or cricket plays, or base balls; they need muscular rest; they should sleep at night, and lie about in the day time under the trees, on the grass, looking up into the sky, with several newspapers or other covering under them, to keep out the dampness from their bodies, eating regularly of plain food, with nothing between; with leisure, walking in the woods, beside the streams and along the road-ways; and when they get home in the autumn, they should at first work leisurely, until the new-made particles of flesh and bone gradually harden and become adapted to severe labor.

The student, the clerk, the book-keeper, the professional man, and all who have sedentary occupations, should

HIE TO THE MOUNTAINS, enter no human dwelling day or night, but camp out and "rough it" for a month; this continuous exposure to out-door air, in hunting and fishing and climbing mountains all the time, will certainly work a wonderful revolution for the better, as to the healthfulness of every one who will try it; there will be no danger of colds; pouring, drenching rains will give no "Rheumatics." Except while actually eating and sleeping, be on the move in some way out of doors, in such a manner as to involve steady and varied muscular activities which will bring into exercise every part of body and brain; the more varied, the more joyous, the better; turn every thing into fun if possible; let uproarious laughter rule the hour; let jest and joke and song and loud huzzaing be the order of the day, at least for an hour after meals, and another hour during each repast. Go and spy out the land, taking a different direction every day from your camping grounds. Endeavor in some way to add to the sum total of human knowledge of actual facts. There is no better place in the world for these things than the Adirondack mountains.

Ten years ago, we advised everybody who could to go there and camp out, relying for their provender on such fish as they could catch, such game as they could bring down, such esculent roots and berries as they might find; and, if they could not replenish their larder in this manner, do without eating until next day—better luck next time. Mr. Murray does not exactly advise this course in his admirable work of "Camp-life in the Adirondacks," but he has made such a delightful volume, which everybody is now reading, that any man or woman either, of even a moderate share of poetry and romance, would think it a happiness to take such a jaunt as the reverend gentleman advises. He tells you all you want to know; why you go to the wilderness, what it costs, how you are to get there; about your outfit, the sporting facilities, healthfulness of camp-life, when to visit the wilderness, which parts are most interesting; and then, there are chapters about the bears, and black flies and mosquitoes, and other bloody animals; and, like a sensible man, he takes it for granted, that ladies are to be in company, and describes their outfit; he knows very well, that the smiles and ambitions, and other things inspired by the presence of woman, make such excursions nineteen hundred and twenty-three thousand times more healthful, happy and delightful, than if only men were in the company. Mr. Murray has done the public an important service in making such a book;—and he has the satisfaction of knowing and feeling (in his pocket) that the said public have called for the volume faster than it could be printed.

### Going Abroad.

A summer excursion to England and the continent is now becoming so common, that not to go abroad, or not to have been abroad, is to be considered out of society; it is not a legitimate conclusion by any means, we want to tell what one person with five hundred paper dollars and three months time can do in the way of sight-seeing in the old world. Those who have incomes of ten thousand dollars a year need not read what follows; this is for persons of moderate means, mainly for energetic and aspiring young men, who are looking towards the professions. All the sums named, after paying the passage across the Atlantic both ways, are in gold. It is to be understood that the following estimates are for those who desire to be economical.

You can take passage in the "Anchor Line of Steamships, No. 6 Bowling-green, New York,"—first cabin, to go and return, from $130 to $160 in paper, according to the size of the state-room; these vessels go to Glasgow, direct, and will send you to Liverpool by steamer; but, if you take a ticket for Liverpool and back, you can, when you get to Glasgow, go to Edinburgh, and they will refund you the cost of passage to Liverpool; when you get to Glasgow stop at the Waverley Hotel,—a strictly temperance establishment; they have a corresponding house in Edinburgh, and in London. You are at once furnished with the printed prices of everything, and you can live comfortably on a paper dollar a day, in either of the cities named. Spend three or four days in Glasgow, and make excursions to the Giant's Causeway, and the birth-place of Burns; go to Dunbarton castle, a few miles down the Clyde, where Wallace was betrayed, and where you are shown his sword, so said. A dollar or two will take you to Edinburgh, that majestic old city, with its immortal histories, from which you can make excursions to Abbottsford, Melrose and Dryburgh Abbeys; to do this and see all the absorbing sights of "Edinbro," the old town and the new, will require a week and ten dollars, besides your board; then take passage to Rotterdam, the mouth of the Rhine, for five or six dollars; stay there a day or two and spend ten dollars in excursions to old Amsterdam, with its famed water streets, and its pile-founded houses; then return to the other "Dam," by way of "the Hague," so memorable in history. Boats leave Rotterdam daily, and on the fourth day arrive at Manheim, beyond which they do not go; they do not travel at night; thus, for five dollars, you will see the whole length of the navigable Rhine, 800 miles; but only one day's travel is historic; in that day you see all its castles and read their wonderful legends. The remainder of the river reminds one of the sameness and flatness of the lower Mississippi; there is no danger of drowning in the Rhine; its average depth is not far from six feet, and its average width, less than five hundred yards; you pass Bonn, and Bingen, and Cologne, and Coblentz, and Mr. Dusseldorf's picture-gallery, and the landings of a great many other interesting places. When you get to Manheim, a half a dollar and half an hour will take you to quaint and quiet old Heidelberg, with its two streets and its glorious castle, and its big barrel which held sixty thousand gallons of wine; but the thirty Germans soon drank it all up; soldiers bombarded the town, and the lightning blazed away at the castle, and barbarous warriors put ever so many tons of powder under it, and did all that was possible to blow it to atoms; and still it stands in part, in defiance of rattling thunders and louder belching artilleries; the magnificent terrace remains, which was the favorite place of promenade for the beautiful princess, the grand-daughter of Mary, Queen of Scots; but, our pen has run astray, we did not intend to write a book of travel, and will only add about Heidelberg, that few who have seen it once, but will many a time in after-life long to see it again; its vine-clad hills, its towering mountains rising upwards two thousand feet away into the skies. Persons visiting Heidelberg will find in Herr Lang, an accommodating host; and, living in his own house, speaking English fluently, as well as other modern languages, himself a traveller, they will be fortunate who find a vacancy in his establishment; he is an intelligent gentleman, courteous in manners, prompt in attentions, and very moderate in charges.

It will be well at this point for the traveller to count how many dollars are in his pocket, and laying by a hundred, he can spend the remainder in excursions to the Black Forest, Frankfort, Dresden, etc., and then take the rail direct for Paris. Fare twelve dollars; remain there ten days, then for London direct in ten or twelve hours, and laying by fifteen dollars to take you to Liverpool, and keep you there a day or two, spend all you have in London, sight-seeing and short excursions. When you get to Liverpool, the "Anchor Line" Steamship Co., will send you to Glasgow without charge, where you can embark any Saturday noon for the United States, which you will be glad enough to see again.

While travelling, purchase for twenty-five cents, Bradshaw's Railway Guide, corrected monthly; it will tell you all the routes of travel, distance, time, cost, the best hotels, second best, printed prices, etc. None should fail of providing himself at New York, before going on board ship, with Harper's Hand-book of Foreign Travel, in morocco flexible binding, with very instructive maps. This should be studied on the voyage; and, before you go to any place, read all that Harper says about it; you will then know what you ought to see, how to see it in the shortest time, and at the least cost. We found it an invaluable guide, correct and reliable on all points, routes, modes of communication, time, distances, best places to stop, etc., etc. We have purposely advised visiting the largest places last, this gives greater interest in the smaller places, and makes the excursion much more enjoyable. To those who have abundant means and time, other routes and modes of travel would be preferable.

After all it would be more profitable to mind and body, as to a large class of persons who have five hundred dollars and three months to spare, to go to San Francisco and back by the Pacific Railway, stopping a day or two at the important stations, making excursions from them in different directions, thus getting an idea of the grandeur and extent of our own country—its resources, its capabilities, the magnificence of its scenery, its mountains and its plains, its rivers, its trees, its lakes, and its water-falls; and on going abroad thereafter, comparisons can be made and conclusions drawn, which will be an unfailing source of interest to the excursionist himself, and to those with whom he may chance to fall into conversation.

